# Retraction: Genetic Manipulation of *Leishmania donovani* to Explore the Involvement of Argininosuccinate Synthase in Oxidative Stress Management

**DOI:** 10.1371/journal.pntd.0011184

**Published:** 2023-03-10

**Authors:** The PLOS Neglected Tropical Diseases Editors

Following the publication of this article [1], concerns were raised regarding similarities between results presented in Figs 3, 4, and S2. Specifically,

- The Fig 3F and S2 Fig panels appear similar.
- The Fig 3F LdAct and S2 Fig LdAct appear similar to the Fig 4C β-actin UNS-S+M results.
- The Fig 4C WT *LdASS* M and S+M results appear similar to the Fig 4C OE *LdASS* L-Arg (200mg/L) and L-Arg(50mg/L) results respectively.
- The Fig 4C WT *LdASS* L-Arg (200mg/L) and L-Arg(50mg/L) results appear similar to the Fig 4C OE UNS and S results respectively.

The first author stated that the Fig 3D, Fig 4C, and S2 Fig results were obtained from different western blot experiments, and that the similarities between the results are due to the same materials being used in these experiments. The underlying data provided for these figures were incomplete and did not resolve the concerns raised with Figs 3, 4, and S2.

The first author stated that original data underlying Figs 2, 5B, 7A, 7B, and some of the raw blot data underlying Figs 3A, 3F, 4C, and 4D, are still available. The original data underlying the results presented in Figs 3G, 4A, 4B, 4E, 4F, 6, 7C, and 7D are no longer available.

In light of the unresolved image concerns that question the reliability of these data, the *PLOS Neglected Tropical Diseases* Editors retract this article.

AHS did not agree with the retraction. AJ, AKG, AM, SD, SS, KA, RS, SV, AK, and PD either did not respond directly or could not be reached.
